# NanoSNP: a progressive and haplotype-aware SNP caller on low-coverage nanopore sequencing data

**DOI:** 10.1093/bioinformatics/btac824

**Published:** 2022-12-22

**Authors:** Neng Huang, Minghua Xu, Fan Nie, Peng Ni, Chuan-Le Xiao, Feng Luo, Jianxin Wang

**Affiliations:** School of Computer Science and Engineering, Central South University, Changsha 410083, China; Hunan Provincial Key Lab on Bioinformatics, Central South University, Changsha 410083, China; School of Computer Science and Engineering, Central South University, Changsha 410083, China; Hunan Provincial Key Lab on Bioinformatics, Central South University, Changsha 410083, China; School of Computer Science and Engineering, Central South University, Changsha 410083, China; Hunan Provincial Key Lab on Bioinformatics, Central South University, Changsha 410083, China; School of Computer Science and Engineering, Central South University, Changsha 410083, China; Hunan Provincial Key Lab on Bioinformatics, Central South University, Changsha 410083, China; State Key Laboratory of Ophthalmology, Zhongshan Ophthalmic Center, Sun Yat-sen University, Guangzhou 510060, China; School of Computing, Clemson University, Clemson, SC 29634, USA; School of Computer Science and Engineering, Central South University, Changsha 410083, China; Hunan Provincial Key Lab on Bioinformatics, Central South University, Changsha 410083, China

## Abstract

**Motivation:**

Oxford Nanopore sequencing has great potential and advantages in population-scale studies. Due to the cost of sequencing, the depth of whole-genome sequencing for per individual sample must be small. However, the existing single nucleotide polymorphism (SNP) callers are aimed at high-coverage Nanopore sequencing reads. Detecting the SNP variants on low-coverage Nanopore sequencing data is still a challenging problem.

**Results:**

We developed a novel deep learning-based SNP calling method, NanoSNP, to identify the SNP sites (excluding short indels) based on low-coverage Nanopore sequencing reads. In this method, we design a multi-step, multi-scale and haplotype-aware SNP detection pipeline. First, the pileup model in NanoSNP utilizes the naive pileup feature to predict a subset of SNP sites with a Bi-long short-term memory (LSTM) network. These SNP sites are phased and used to divide the low-coverage Nanopore reads into different haplotypes. Finally, the long-range haplotype feature and short-range pileup feature are extracted from each haplotype. The haplotype model combines two features and predicts the genotype for the candidate site using a Bi-LSTM network. To evaluate the performance of NanoSNP, we compared NanoSNP with Clair, Clair3, Pepper-DeepVariant and NanoCaller on the low-coverage (∼16×) Nanopore sequencing reads. We also performed cross-genome testing on six human genomes HG002–HG007, respectively. Comprehensive experiments demonstrate that NanoSNP outperforms Clair, Pepper-DeepVariant and NanoCaller in identifying SNPs on low-coverage Nanopore sequencing data, including the difficult-to-map regions and major histocompatibility complex regions in the human genome. NanoSNP is comparable to Clair3 when the coverage exceeds 16×.

**Availability and implementation:**

https://github.com/huangnengCSU/NanoSNP.git.

**Supplementary information:**

Supplementary data are available at *Bioinformatics* online.

## 1 Introduction

Single nucleotide polymorphisms, commonly referred to as SNPs, are human’s most common genetic variation. SNPs are widespread in a person’s DNA, with an average of one SNP occurring in every 1000 nucleotides. Currently, more than 100 million human SNPs have been identified worldwide (1000 Genomes Project Consortium *et al.*, 2015). SNPs may help predict an individual’s response to certain drugs, susceptibility to environmental factors and risk of developing specific diseases (Shastry, 2007). SNPs can also track the inheritance of disease genes in families (Reshef *et al.*, 2018). Identification of SNPs has important implications for clinical applications and genetic studies.

Next-generation sequencing (NGS) has the advantages of low cost and high per-base accuracy. SNP identification on Illumina short-read sequencing data has been well practiced. GATK (DePristo *et al.*, 2011) uses logistic regression models, hidden Markov models (HMM) and Naive Bayes models to identify SNP sites, and then uses Gaussian mixture models to remove false-positive sites. DeepVariant (Poplin *et al.*, 2018) is a deep learning-based SNP caller that calls genetic variation from aligned Illumina sequencing reads by learning statistical relationships between images of reads pileups around putative variant and actual variant calls. However, due to the fundamental limitations of short reads, the sequencing reads frequently fail to align unambiguously in the repetitive regions, resulting in erroneous SNP calls.

Third-generation sequencing technologies (TGS), including PacBio Biosciences (PacBio) and Oxford Nanopore Technologies (ONT), have made significant breakthroughs in a series of genomics studies (Jain *et al.*, 2018; Chen *et al.*, 2021; Ni *et al.*, 2021). Compared with the NGS technologies, the TGS technologies have the advantages of long read length, no PCR amplification and no GC bias. When long reads span repetitive elements, reads can be unambiguously aligned to unique positions in the genome. The Telomere-to-Telomere (T2T) Consortium leverages the additional capabilities of long reads and short reads to finish the first truly complete 3.055 billion base pairs of sequence in the human genome, representing the largest improvement in the human reference genome since its initial release (Nurk *et al.*, 2021).

PacBio offers a sequencing mode called circular consensus sequencing. The resulting HiFi reads have comparable per-base accuracy (99.8%) to Illumina and read lengths of 15–20 kb (Wenger *et al.*, 2019). Due to high per-base accuracy, the SNP identification with DeepVariant on 35× HiFi reads can reach an F1 score of 99.9% (Poplin *et al.*, 2018). The main innovation of Nanopore sequencing is to measure the current changes as single-stranded DNA/RNA goes through a protein nanopore. The current signals are transformed into DNA or RNA sequences by basecalling tools. Nanopore sequencing provides the longest read length of at most 4 Mb (Payne *et al.*, 2019). However, the high median error rate of 6–15% for R9.4 Nanopore reads makes SNP identification challenging (Wang *et al.*, 2021).

Recently, many SNP detection tools have been developed for Nanopore sequencing data, such as Clairvoyante (Luo *et al.*, 2018), Clair (Luo *et al.*, 2020), Clair3 (Zheng *et al.*, 2021), NanoCaller (Ahsan *et al.*, 2021) and Pepper-DeepVariant (Shafin *et al.*, 2021). Clairvoyante is a deep-learning approach to identify SNPs by using a multi-task convolution neural network. Clair is a neural network-based SNP detection tool consisting of two bidirectional long short-term memory layer (LSTM) layers and three feed-forward layers. Clair is the successor to Clairvoyante, and both use the pileup data from reads-to-reference alignment to predict the zygosity and the alternative allele of the candidate SNP site. On the Nanopore dataset with coverage 60×, the performance of SNP identification with Clair can reach an F1 score of 98.5% (Luo *et al.*, 2020). Clair3 (Zheng *et al.*, 2021) is the third generation of Clairvoyante and Clair. Clair3 predicts most variant candidates with pileup calling as Clair and applies full-alignment calling to handle complicated candidates. In full-alignment calling, Clair3 combines the reads alignment and phasing information at the candidate site to form an image of the candidate and uses a residual neural network (ResNet) to predict the genotype and zygosity. Clair3 has superior accuracy for SNP detection of low-coverage Nanopore sequencing reads. NanoCaller solely uses long-range haplotype information from reads alignment for SNP calling. Compared to local pileup information, haplotype structure can span hundreds or even thousands of bases away from the candidate site. In NanoCaller, the haplotype information is fed into a deep convolution neural network for predicting the zygosity and the alternate allele of the candidate site. On 50× Nanopore sequencing reads, the performance of SNP calling with NanoCaller can reach an F1 score of 98.6% (Ahsan *et al.*, 2021). Pepper-DeepVariant is a haplotype-aware pipeline for identifying SNPs against a reference genome with Nanopore sequencing reads. The pipeline employs several methods to generate highly accurate variant calls. PEPPER-SNP adopts a recurrent neural network to find candidate SNPs from pileup data of reads alignment. Then, Margin uses an HMM model to phase the alignment file according to the candidate SNPs. PEPPER-HP has a similar network structure as PEPPER-SNP but predicts the candidate SNP sites with the pileup data of phased alignment files. Finally, the candidate SNPs are verified by DeepVariant, which is a bigger neural network with more parameters. Combining PEPPER with DeepVariant allows a faster network to find the candidates and a larger network to achieve high accuracy. When the sequencing reads have at least 50× coverages, the performance of SNP calling with Pepper-DeepVariant can reach an F1 score of 99.6% (Shafin *et al.*, 2021).

The aforementioned SNP callers designed for Nanopore sequencing reads have high accuracy, but there are still limitations. Currently, most SNP callers require high-coverage sequencing reads. The sequencing reads with low coverage will reduce the accuracy of SNP calling. Since the human genome has about 3 billion base pairs, the sequencing cost of high-coverage Nanopore sequencing cannot be ignored. The cheapest option for generating long reads is the ONT PromethION platform, which can generate 100–150 Gbp of data per flowcell. In a large population study with 2500 samples, assuming a diploid genome with a similar size to a haploid human genome (3.2 Gbp), it will cost approximately $1 375 000 to obtain the Nanopore dataset with 50× sequencing coverage. Therefore, it is very urgent to improve the accuracy of identifying SNPs on low-coverage sequencing reads. The difficulty of SNP identification for low-coverage Nanopore sequencing is that the error rate of Nanopore reads is high, and the coverage of reads is insufficient to determine whether the base difference between reads and reference is a sequencing error or an actual variation. For the lack of SNP information in low-coverage Nanopore reads, phasing can divide the sequencing reads into different haplotypes, which provides additional information. Phasing information can help to improve the accuracy of SNP detection. Likewise, family structure data can also provide additional information for SNP detection, which can help to increase the sensitivity of low-coverage SNP detection.

This article presents a neural network-based SNP caller named NanoSNP for low-coverage Nanopore sequencing data. To overcome the lack of information on SNP detection with low-coverage Nanopore sequencing data, we successively apply two prediction models to identify SNPs from different perspectives. We divided the main process into the following three steps. We first identified a set of SNP sites with the pileup model based on the read pileups of aligned reads. Then the SNP sites are phased and used to divide the Nanopore sequencing reads into different haplotypes. Finally, the high-quality SNPs predicted in the pileup model and the reads of each haplotype are used to construct the haplotype features of candidate sites. We use the haplotype model to combine the long-range haplotype feature and short-range pileup feature from each haplotype and predict the genotype of each candidate SNP. We train NanoSNP on the dataset of HG001 as Clair and NanoCaller. On the six low-coverage (∼16×) Nanopore sequencing datasets of human genomes HG002-HG007, NanoSNP has the highest precision score and second highest recall and F1 score on each dataset compared to Clair, Clair3, Pepper-DeepVariant and NanoCaller. When evaluating the SNPs in the difficult-to-map regions, NanoSNP outperforms the compared Nanopore SNP callers on each genome.

## 2 Materials and methods

### 2.1 NanoSNP framework for low-coverage nanopore SNP calling

**Fig. 1.**
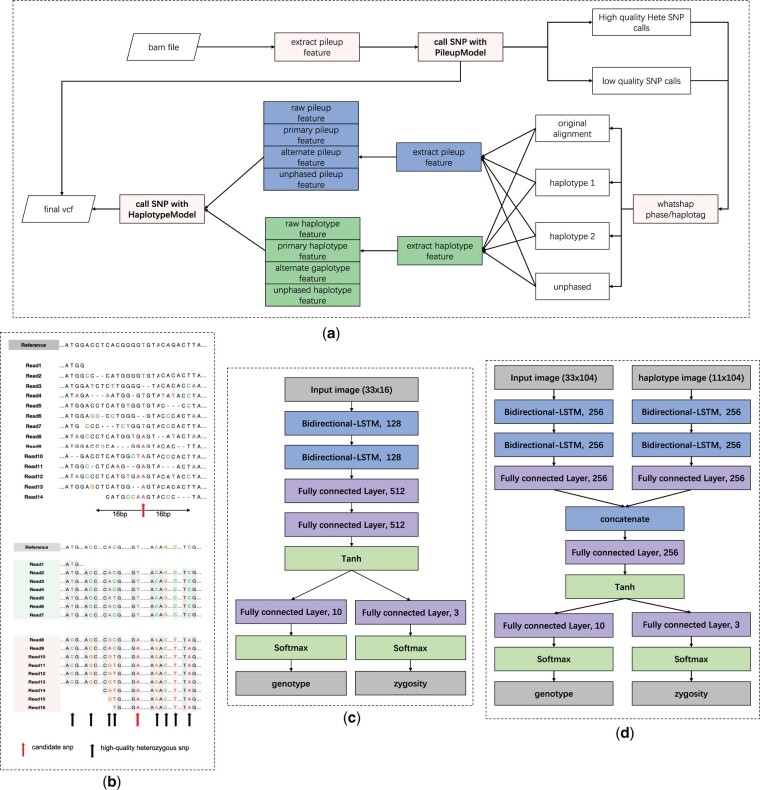
(**a**) The workflow of SNP caller NanoSNP. (**b**) The pileup information (upper) and the haplotype information (lower) of the alignment. (**c**) The network architecture of the pileup model in NanoSNP. (**d**) The network architecture of the haplotype model in NanoSNP

The workflow of NanoSNP is shown in Figure 1a. First, the Nanopore reads are aligned to the reference genome using Minimap2 (Li, 2018). The candidate site is determined by the coverage and the alternative allele frequency at a specific genomic position. Then, the pileup feature is extracted from the reads-to-reference alignment at each candidate site. NanoSNP uses a recurrent neural network-based (Hochreiter and Schmidhuber, 1997) pileup model for the first round of SNP detection. From the results of the pileup model, the low-quality predictions and the high-quality heterozygous SNP sites are selected. The low-quality predictions will enter the second round of SNP detection. The high-quality heterozygous SNP sites are phased by WhatsHap (Martin *et al.*, 2016). According to the phasing result, the reads alignment is divided into different haplotypes. On each haplotype, NanoSNP extracts the long-range haplotype feature and the short-range pileup feature of each candidate SNP site as shown in Figure 1b. These two types of features are fed into the haplotype model of NanoSNP to perform the second round of SNP detection. The haplotype model consists of two bidirectional LSTM networks. Finally, the results of two rounds of SNP calling are merged.

**Search for candidate SNP.** Pileup format is a text-based format for summarizing the base calls of aligned reads to a reference sequence. We use Samtools to generate the pileup data from reads-to-reference alignment. The candidate site is determined by the depth and alternative allele frequency at a specific genomic position. Given the pileup data and the reference base *B_r_* at site *b*, the alternative allele frequency *f_b_* is calculated as follows:

$$f_{b}=\frac{\max\{\text{num}\;\text{of}\;\text{base}\;B\;\text{at}\;\text{site}\;b|B\in \{A,C,G,T,I,D\}\setminus B_{r}\}}{\text{depth}\;\text{at}\;\text{site}\;b}.$$

If the depth is greater than *d* and the alternative allele frequency is greater than *e*, genomic position *b* is considered a candidate SNP site. We set the default thresholds of depth and allele frequency to 6 and 12%, respectively. Smaller values of these two thresholds will increase the accuracy of SNP calling but will also increase the number of candidate sites and thus increase computational resources.

**Generation of pileup image in pileup model.** For a given candidate site *b* and a window size *w*, we extract the read fragments aligned to the region [b−w,b+w] on the reference. We select the forward reads first and extract the pileup feature ($C_{p}[A],C_{p}[C],C_{p}[G],C_{p}[T],C_{p}[I],C_{p}[D],\mathrm{IMA}X_{p},\mathrm{DMA}X_{p}$) for each position p∈[b−w,b+w]. Here, $C_{p}[B]=||\{R_{i}[p]=B,i\in \{1,2,\ldots ,m\}\}||,B\in \{A,C,G,T,I,D\}$. $\left\{R_{1},\ldots ,R_{i},\ldots ,R_{m}\right\}$ are *m* forward reads. IMAX_*p*_ is the maximum length of inserted fragments at position *p* and DMAX_*p*_ is the maximum length of deleted fragments at position *p*. The processing of the reverse reads is similar to that of the forward reads. We construct two feature matrices from forward reads and reverse reads, respectively. Each feature matrix is in size of [(2w+1)×8]. The whole pileup image *P_b_* of the candidate site *b* has the shape of [(2w+1)×16] by concatenating the two feature matrices. Referring to other pileup-based SNP calling methods such as Clair, we set the default value of *w* to 16.

**Pileup prediction model.** Given the pileup image *P_b_* of the candidate site *b*, NanoSNP has a two-tasks recurrent neural network to predict the zygosity and genotype. The number of zygosities is 3, including homozygous reference (0/0), homozygous variant (1/1) and heterozygous variant (0/1). The number of genotypes is 21, including ‘AA, AC, AG, AT, CC, CG, CT, GG, GT, TT, AI, AD, CI, CD, GI, GD, TI, TD, II, DD, ID’. The architecture of the neural network is shown in Figure 1c. The neural network of the pileup model consists of two bidirectional LSTM layers followed by two fully connected layers. The hidden size of both bidirectional LSTM layers is 128, and the output size of the fully connected layer is 512. To predict the zygosity and genotype of the candidate SNP, we extract the middle time step in the sequence of tensor after the tanh activation. Then, the fully connected layer and the softmax activation are used to calculate two types of probabilities. Let $P_{g}(L1)$ be the probability of the most likely class *L*1 in genotype prediction and $P_{z}(L2)$ be the probability of the most likely class *L*2 in zygosity prediction. With the probabilities, we can calculate the quality scores of the genotype prediction and the zygosity prediction as $10-10\times \ln10\times \ln\frac{1-P}{P},P\in \{P_{g}(L1),P_{z}(L2)\}$. The final quality score is the smaller of the genotype score and the zygosity score. During training, we apply cross-entropy loss and label smoothing to calculate the losses of the two tasks, and the total loss is obtained by adding the two losses. The optimizer in the pileup model is Adam combined with the lookahead strategy (Wright and Demeure, 2021). The batch size equals 2000, the learning rate equals 0.0001 and the number of training epochs equals 200. The training of the pileup model takes around 80 h on a GPU device.

**Haplotyping.** Phasing for long-read sequencing improves SNP calling, structural variant calling and genome assembly (Patterson *et al.*, 2015; Huddleston *et al.*, 2017; Porubsky *et al.*, 2021). On the low-coverage sequencing reads, when the alternative allele frequency of the SNP and the sequencing error rate are close to each other, it is difficult to identify actual SNP by local pileup information. So, we try to phase the reads to distinguish sequencing errors and SNPs in different haplotypes. We have identified a subset of SNPs from the candidate sites with the pileup model of NanoSNP. Then, the high-quality heterozygous SNP sites are selected from the output of the pileup model and these heterozygous SNPs are phased by WhatsHap (Martin *et al.*, 2016). Afterward, we divide the alignment bam file into different haplotypes based on the phased SNPs with the command ‘WhatsHap haplotag’. Each phased bam stores the sequencing reads from the same haplotype.

**Generation of haplotype image and pileup image in haplotype model.** For a set of reads aligned to the reference, we represent the reads-to-reference alignment, the base quality of each read and the mapping quality of each read with the following matrices *A*, *Q* and *M*:

$$A=\left[\begin{array}{ccccccc} R_{0}[0] & R_{0}[1] & \ldots & R_{0}[j] & \ldots & R_{0}[n-1] & R_{0}[n]\\ R_{1}[0] & R_{1}[1] & \ldots & R_{1}[j] & \ldots & R_{1}[n-1] & R_{1}[n]\\ & & & \ldots & & & \\ R_{i}[0] & R_{i}[1] & \ldots & R_{i}[j] & \ldots & R_{i}[n-1] & R_{i}[n]\\ & & & \ldots & & & \\ R_{m}[0] & R_{m}[1] & \ldots & R_{m}[j] & \ldots & R_{m}[n-1] & R_{m}[n] \end{array}\right],$$

$$Q=\left[\begin{array}{ccccccc} Q_{0}[0] & Q_{0}[1] & \ldots & Q_{0}[j] & \ldots & Q_{0}[n-1] & Q_{0}[n]\\ Q_{1}[0] & Q_{1}[1] & \ldots & Q_{1}[j] & \ldots & Q_{1}[n-1] & Q_{1}[n]\\ & & & \ldots & & & \\ Q_{i}[0] & Q_{i}[1] & \ldots & Q_{i}[j] & \ldots & Q_{i}[n-1] & Q_{i}[n]\\ & & & \ldots & & & \\ Q_{m}[0] & Q_{m}[1] & \ldots & Q_{m}[j] & \ldots & Q_{m}[n-1] & Q_{m}[n] \end{array}\right],$$

$$M=\left[\begin{array}{ccccccc} M_{0}[0] & M_{0}[1] & \ldots & M_{0}[j] & \ldots & M_{0}[n-1] & M_{0}[n]\\ M_{1}[0] & M_{1}[1] & \ldots & M_{1}[j] & \ldots & M_{1}[n-1] & M_{1}[n]\\ & & & \ldots & & & \\ M_{i}[0] & M_{i}[1] & \ldots & M_{i}[j] & \ldots & M_{i}[n-1] & M_{i}[n]\\ & & & \ldots & & & \\ M_{m}[0] & M_{m}[1] & \ldots & M_{m}[j] & \ldots & M_{m}[n-1] & M_{m}[n] \end{array}\right].$$

Here, $R_{i}[j],\;Q_{i}[j]$ and $M_{i}[j]$ are the aligned base, base quality and mapping quality of a given read at the reference position *j*. The number of aligned reads is *m *+* *1 and the size of the region in the reference is *n *+* *1.

For a set of aligned reads, we designed three kinds of features, which are nucleotide distribution, base quality distribution and mapping quality distribution. The details of three kinds of features are described below.

1. Nucleotide distribution: the number of different nucleotides $C_{j}^{B}$ and the frequency of different nucleotide $F_{j}^{B}$ at the given reference position. For each nucleotide B∈{A,C,G,T,D}, the computations of the features are as follows:
  $$g_{i,j}^{B}=\{\begin{array}{cc} 1 & \text{if}\;\;R_{i}[j]=B,\\ 0 & \text{otherwise}. \end{array}$$
  $$C_{j}^{B}=\sum_{i=1}^{m}R_{i}[j]*g_{i,j}^{B},$$
  $$F_{j}^{B}=\frac{C_{j}^{B}}{C_{j}^{A}+C_{j}^{C}+C_{j}^{G}+C_{j}^{T}+C_{j}^{D}}.$$
2. Base quality distribution: the sum of base qualities of different nucleotides $Q_{j}^{B}$ and the mean value of base qualities of different nucleotides $U_{j}^{B}$ at the given reference position. For each nucleotide other than the deletion B∈{A,C,G,T}, we calculate the features as follows:
  $$Q_{j}^{B}=\sum_{i=1}^{m}Q_{i}[j]*g_{i,j}^{B},$$
  $$U_{j}^{B}=\frac{Q_{j}^{B}}{\sum_{i=1}^{m}g_{i,j}^{B}}.$$
3. Mapping quality distribution: the sum of mapping qualities of different nucleotides $M_{j}^{B}$ and the mean value of mapping qualities of different nucleotides $V_{j}^{B}$ at the given reference position. For each nucleotide B∈{A,C,G,T}, the features of the mapping qualities are computed as follows:
  $$M_{j}^{B}=\sum_{i=1}^{m}M_{i}[j]*g_{i,j}^{B},$$
  $$V_{j}^{B}=\frac{M_{j}^{B}}{\sum_{i=1}^{m}g_{i,j}^{B}}.$$

Then, we concatenate the three aforementioned types of features into a flattened tensor *T_j_* as a combined feature tensor at each position *j* of the reference as follows:

$$\begin{array}{cc} T_{j}= & \left[C_{j}^{A},C_{j}^{C},C_{j}^{G},C_{j}^{T},C_{j}^{D}\right]\\ + & \left[F_{j}^{A},F_{j}^{C},F_{j}^{G},F_{j}^{T},F_{j}^{D}\right]\\ + & \left[Q_{j}^{A},Q_{j}^{C},Q_{j}^{G},Q_{j}^{T}\right]\\ + & \left[U_{j}^{A},U_{j}^{C},U_{j}^{G},U_{j}^{T}\right]\\ + & \left[M_{j}^{A},M_{j}^{C},M_{j}^{G},M_{j}^{T}\right]\\ + & [V_{j}^{A},V_{j}^{C},V_{j}^{G},V_{j}^{T}]. \end{array}$$

The local pileup extracts the reads alignment information from the adjacent region around the candidate SNP site. The long range haplotype feature can take full advantage of the long read length of Nanopore reads to collect the alignment information in the region that is far away from the candidate SNP site. For a given candidate SNP site *b* and flanking size *w*, the combined feature *T_j_* at each position in the region [b−w,b+w] forms the local pileup image *P_b_* with the shape of (2w+1)×26 like:

$$P_{b}=\left[\begin{array}{c} T_{b-w};\ldots ;T_{b-1};T_{b};T_{b+1};\ldots ;T_{b+w} \end{array}\right].$$

In order to extract the long-range haplotype information, we select the subset of the high-quality heterozygous SNP sites around the candidate SNP site. Let $\left\{l_{1},l_{2},\ldots ,l_{t}\right\}$ be *t* high-quality heterozygous sites on the left side of the candidate SNP site *b* and $\left\{r_{1},r_{2},\ldots ,r_{t}\right\}$ be *t* high-quality heterozygous sites on the right side of the candidate site. For a candidate SNP site *b*, the combined feature *T_j_* at each position in the sites $\left\{l_{1},l_{2},\ldots ,l_{t},b,r_{1},r_{2},\ldots ,r_{t}\right\}$ forms the long-range haplotype image *H_b_* with the shape of (2t+1)×26 like:

$$H_{b}=\left[\begin{array}{c} T_{l_{1}};\ldots ;T_{l_{t}};T_{b};T_{r_{1}};\ldots ;T_{r_{t}} \end{array}\right].$$

In the haplotype model, to extract the SNP information from different haplotypes, we divide the original alignment into three parts according to the phasing result, which is the alignment of haplotype 1, the alignment of haplotype 2 and the unphased alignment. For each candidate SNP site *b*, we extract four pileup images ($P_{b}^{\text{ori}},P_{b}^{\text{hap}1},P_{b}^{\text{hap}2},P_{b}^{\mathrm{unp}}$) and four haplotype images ($H_{b}^{\text{ori}},H_{b}^{\text{hap}1},H_{b}^{\text{hap}2},H_{b}^{\mathrm{unp}}$) from the original alignment and the three sub-alignments, respectively. Finally, the four pileup images are concatenated from the second dimension to form the complete pileup image with the size of (2w+1)×104. The four haplotype images are processed similarly to obtain the complete haplotype image with the size of (2t+1)×104. Here, the flanking size *w* of the pileup image has the same value as the flanking size in the pileup model which equals 16. The number of high quality heterozygous sites *t* on either side of a candidate site affects the number of reads which cover all the heterozygous sites simultaneously. When the number of heterozygous sites is big, the number of aligned reads will be insufficient. When the number of heterozygous sites is small, the haplotype feature cannot provide long range information. So we set the quality threshold of high quality heterozygous sites to 14 according to Supplementary Table S1 and the number of the selected heterozygous sites *t* of each side of the candidate is set to 5. In Supplementary Table S2, we evaluate the effect of the pileup image and the haplotype image in the haplotype model. Neither the pileup image nor the haplotype image alone can achieve the best performance. The model achieves the highest F1 score when using the pileup image and the haplotype image together.

**Haplotype prediction model.** In NanoSNP, the haplotype model consists of two modules, the pileup image processing module and the haplotype image processing module. The architecture of the haplotype model is shown in Figure 1d. Both modules apply the same neural network architecture, which consists of two bidirectional LSTM layers followed by a fully connected layer. Since both the pileup image and the haplotype image are essentially the combination of alignment features at different positions of the reference, the bidirectional LSTM layers are used to capture the feature correlation before and after the candidate SNP site. Behind the pileup processing module and the haplotype processing module, there is a fully connected layer combining the outputs of the two modules. Finally, two fully connected layers take the combined outputs as the input and predict the genotype and zygosity of the candidate SNP site, respectively.

The number of genotypes is 10, including ‘AA, AC, AG, AT, CC, CG, CT, GG, GT, TT’. The number of zygosities is 3, including homozygous reference, homozygous variant and heterozygous variant. During training, the parameters are optimized by Adam combined with the lookahead strategy (Wright and Demeure, 2021). Meanwhile, we employ label smoothing of value 0.1 to improve the robustness of the model. The initial learning rate is set to be 0.00001. The batch size equals 512 and the number of training epochs equals 30. The training of the haplotype model takes around 45 h with a GPU device. During inference, we select the genotype class and zygosity class with the highest probabilities as the output of the haplotype model. Based on the probabilities, the quality score of the prediction is calculated as $10-10\times \ln10\times \ln\frac{1-P}{P},P\in \{P_{g}(L1),P_{z}(L2)\}$. $P_{g}(L1)$ is the probability of the most likely class *L*1 in genotype prediction and $P_{z}(L2)$ is the probability of the most likely class *L*2 in zygosity prediction. The smaller of the genotype score and the zygosity score is used as the score of the prediction by the haplotype model.

**Merging SNP calls from the haplotype model and the pileup model.** The final SNP calls of NanoSNP are obtained by combining the predictions of the pileup model and that of the haplotype model. If the SNP site identified by the haplotype model has a quality score greater than a quality threshold *δ*, NanoSNP will add the SNP site to the final VCF file. On the contrary, if the quality score is less than *δ*, the confidence of the SNP call by the haplotype model is not high enough. Then NanoSNP checks the prediction of the pileup model at the same position. If the quality score is greater than *δ*, the SNP site identified by the pileup model will be added to the final VCF file. If the quality score is also less than *δ*, the SNP call is considered a false-positive prediction and will be discarded. By testing different values of *δ* in Supplementary Table S3, we set the default quality threshold *δ* to 14. The haplotype information used in NanoSNP is not written to the output VCF, but the genotype and zygosity predicted by the network are written to the output VCF.

**Algorithmic comparison of NanoSNP and Clair3.** NanoSNP and Clair3 have similarities in the process of SNP identification. The SNP caller first uses the naive pileup feature to perform the first round of SNP detection. The detected SNPs are then used to generate the phasing information. Afterward, the SNP caller combines the phasing information with the alignment information for the second round of SNP detection. Finally, the results of two rounds of SNP calling are merged. Actually, NanoSNP and Clair3 have many differences in implementation details. In the pileup model, the architecture of NanoSNP’s recurrent network differs from that of Clair3’s recurrent neural network. In the haplotype model, the feature matrix of NanoSNP is completely different from the full-alignment image of Clair3. NanoSNP uses complex feature extraction strategies to obtain the distribution of different bases, the base quality distribution of different bases, and the mapping quality distribution of different bases at each position of the reference. The features at the position around the candidate site form the local pileup feature, and the features at the position of the high-quality heterozygous sites around the candidate site form the long-range haplotype feature. And NanoSNP extracts the features from both the alignment before WhatsHap phasing and the phased alignment. Clair3 directly uses the phased local sequence alignment around the candidate SNP site as an feature image. The neural networks of the second round of SNP calling in NanoSNP and Clair3 are quite different. The neural network of the haplotype model in NanoSNP is two individual Bi-LSTM networks followed by several fully connected layers. The neural network of full-alignment model in Clair3 is a residual neural network with three standard residual blocks.

## 3 Experiments and results

### 3.1 Training and testing datasets

We train NanoSNP on HG001 Nanopore sequencing data subsampled at different coverage values (25×, 50× and 75×). The training dataset is a mixture of the three datasets including the 25× HG001 dataset, 50× HG001 dataset and 75× HG001 dataset. The raw Nanopore reads are basecalled by Guppy v4.2.2. We divide the dataset into two parts, chromosomes 1–19 are used for training, and chromosomes 20–22 are used for validation. The six testing datasets are made from Nanopore sequencing reads of human genomes HG002–HG007. The details of each human dataset are given in Supplementary Table S4. In all the experiments, the sequencing reads are aligned to GRCh38 with Minimap2 (Li, 2018). Each human sample uses the GIAB variants v3.3.2 (Zook *et al.*, 2019) as the benchmark datasets.

### 3.2 SNP-calling performance on different coverages of low-coverage nanopore sequencing reads

The datasets of two human genomes HG002 and HG003 are used to evaluate the performance of several SNP callers on low-coverage Nanopore sequencing data with different coverage values. We randomly sampled four subsets from each genome of HG002 and HG003, respectively. The coverages of the subsets are 10×, 13×, 16×, 19×, 22× and 25×. The five Nanopore SNP callers are Clair, Clair3, Pepper-DeepVariant, NanoCaller and NanoSNP. Clair, Clair3, NanoCaller and NanoSNP were all trained on the genome HG001. The Nanopore sequencing reads of HG001 were basecalled by Guppy v4.2.2. Pepper-DeepVariant was trained on HG002 Nanopore sequencing data, which was basecalled by Guppy v4.2.2. During inference, we obtained the alignment bam files by aligning the sequencing reads of HG002 and HG003 to the reference GRCh38, respectively. Then, each SNP caller took the bam file as input and detected the SNPs with the default parameters for Nanopore data. The SNP calls of each method were evaluated with the haplotype comparison tool hay.py (https://github.com/Illumina/hap.py) against the GIAB truth variants dataset (v3.3.2).

**Fig. 2.**
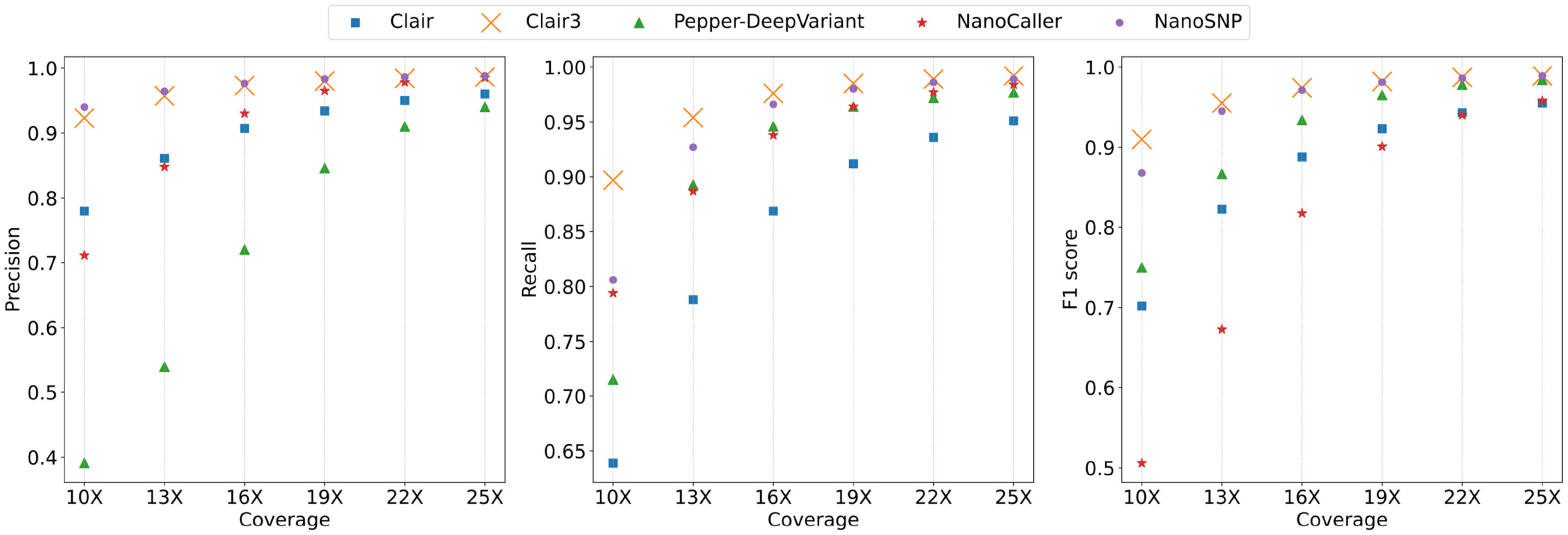
Precision, Recall, and F1 score of the SNP calls on HG002 low-coverage Nanopore datasets with different coverages. The SNP callers are Clair, Clair3, Pepper-DeepVariant, NanoCaller and NanoSNP

The SNP calls of several SNP callers on HG002 Nanopore datasets with different coverages (10×, 13×, 16×, 19×, 22× and 25×) are shown in Figure 2. NanoSNP and Clair3 outperform Clair, Pepper-DeepVariant and NanoCaller when the reads coverage is low. In each dataset, NanoSNP has the highest precision and second highest recall and F1 score. Clair3 has the highest recall and F1 score. As the sequencing coverage increases from 10× to 25×, the precision of NanoSNP increases from 94.0 to 98.8%, the recall increases from 80.6 to 98.9%, and the F1 score increases from 86.8 to 98.9%. When the coverage of the dataset is greater than 16×, the F1 score of NanoSNP exceeds 95% and the performance of NanoSNP is close to that of Clair3. When sequencing coverage is equal to 25×, the F1 score of Pepper-DeepVariant is close to that of NanoSNP and Clair3. Since the F1 score of each SNP caller on the dataset at 10× coverage is low, the SNP calls cannot be used in downstream analysis. Hence, we use the Nanopore datasets at 16× coverage to evaluate different SNP callers. TP is the number of SNPs correctly detected by an SNP caller, FP is the number of predicted SNPs which are not in benchmark and FN is the number of SNPs in benchmark which are not detected by an SNP caller. We cluster the FP/FN calls preduced by NanoSNP on the dataset of 16× HG002 reads (Supplementary Text S2). The FP calls are clustered in the large duplications of the genome and the FN calls are rarely clustered. The main reason for the FN calls is low read coverage. The coverage distributions at TP/FP/FN sites predicted by different SNP callers are shown in Supplementary Text S3. The coverage of FN sites in NanoSNP is lower than that of TP and FP sites, and the coverage of TP sites is slightly higher than that of FP sites. In Supplementary Text S4, we show the reads alignment of the SNP site that is correctly identified by NanoSNP but incorrectly identified by all other tools.

For the HG002 genome, we evaluate the accuracy of different SNP callers in non-diploid regions. We select the regions containing heterozygous deletions and duplications as non-diploid regions from the public structure variation dataset for HG002. We then evaluate the SNP sites falling in the non-diploid regions. From Supplementary Table S5, NanoSNP and Clair3 have higher accuracy than Clair, Pepper-DeepVariant and NanoCaller. The performance of NanoSNP and Clair3 is very close. There are a total of 2422 SNPs in the non-diploid regions, of which 2355 are identified by Clair3 and 2342 are detected by NanoSNP. Clair3 identifies 13 more SNPs than NanoSNP, and NanoSNP has 3 fewer false positive SNP than Clair3.

The dataset of the genome HG003 is new to these five SNP callers, each tool has not been trained on this genome. The evaluation of five SNP callers on HG003 Nanopore datasets at different coverages is shown in Supplementary Table S6. On six datasets, NanoSNP obtains the highest precisions in five SNP callers. Clair3 has the highest recalls and F1 scores. When the reads coverage is greater than 16×, the F1 scores of NanoSNP are close to that of Clair3. At the coverage of 16×, 19×, 22× and 25×, the F1 scores of NanoSNP are 97.1%, 98.1%, 98.6% and 98.8%. Clair3 obtains the F1 scores of 97.4%, 98.2%, 98.7% and 98.9%. The F1 scores of NanoSNP and Clair3 are much higher than the F1 scores of Clair (89.3%, 92.6%, 94.5% and 95.6%), Pepper-DeepVariant (92.8%, 96.3%, 97.7% and 98.4%) and NanoCaller (82.6%, 90.3%, 93.9% and 95.6%). The F1 score of NanoSNP gets improved by 8.7%, 4.6% and 17.6% compared to that of Clair, Pepper-DeepVariant and NanoCaller, respectively at the coverage of 16×.

On low-coverage Nanopore sequencing data of HG002 and HG003, NanoSNP outperforms Clair, Pepper-DeepVariant and NanoCaller, and the performance of NanoSNP is close to that of Clair3. We then perform cross-reference validation on NanoSNP. NanoSNP is trained on HG001 and GRCh38, we test the accuracy of NanoSNP on HG002, HG003 sequencing data and GRCh37 reference. Both the sequencing data and reference genome are new to the model of NanoSNP. From Supplementary Table S7, the accuracy of NanoSNP on the GRCh37 reference is slightly higher than that on the GRCh38 reference, which can indicate that NanoSNP has good robustness for SNP detection on different references.

### 3.3 SNP-calling performance on six human samples low-coverage nanopore data

In real-world scenarios, SNP callers usually need to identify the SNPs in different human genomes, which requires the method to perform stably on different genomes. We used six human genomes (HG002–HG007) to perform cross-genome testing of five SNP callers Clair, Clair3, NanoCaller, Pepper-DeepVariant and NanoSNP. We used Samtools to randomly subsample the Nanopore sequencing data of each genome at low coverage 16×. We aligned the Nanopore reads to the reference GRCh38 with Minimap2 and then fed the alignment bam file into each SNP caller. The truth variants datasets are from GIAB variants v3.3.2.

**Fig. 3.**
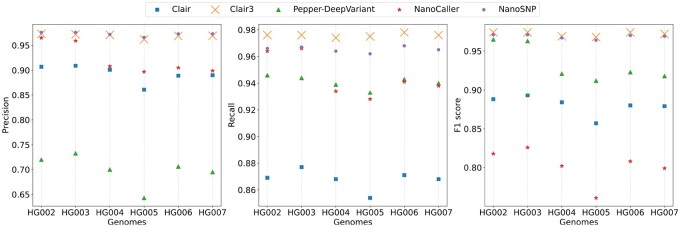
Cross-genome testing of Clair, Clair3, Pepper-DeepVariant, NanoCaller and NanoSNP on low-coverage (16×) Nanopore sequencing datasets of genomes HG002-HG007

The evaluation of different SNP callers on low-coverage Nanopore sequencing reads of six human genomes is shown in Figure 3. On the dataset of Ashkenazim Trio (HG002, HG003 and HG004), NanoSNP has the highest precisions among the five SNP callers. Clair3 has the highest recalls (97.6%, 97.6% and 97.4%) and F1 scores (97.4%, 97.4% and 96.9%) in five SNP callers and NanoSNP has the second-highest recalls (96.6%, 96.7% and 96.4%) and F1 scores (97.1%, 97.1% and 96.7%). The F1 scores of other SNP callers including Clair, Pepper-DeepVariant and NanoCaller are significantly lower than the F1 scores of NanoSNP and Clair3. The comparison of TP/FP/FN calls of NanoSNP and Clair3 on the dataset of HG003 is shown in Supplementary Text S5.

On the dataset of Chinese Trio (HG005, HG006 and HG007), NanoSNP achieves the highest precisions (96.6%, 97.3% and 97.3%) in five SNP callers. The F1 scores of NanoSNP are 96.4%, 97.0% and 96.9%. Clair3 obtains the highest recalls and F1 scores. The F1 scores of Clair3 are 96.8%, 97.4% and 97.2%. The F1 socres of other SNP callers are much lower than the F1 scores of NanoSNP and Clair3. The F1 scores of Clair are 85.7%, 88.0% and 87.9%. The F1 scores of Pepper-DeepVariant are 91.2%, 92.3% and 91.8%. The F1 scores of NanoCaller are 76.1%, 80.8% and 79.9%.

We also evaluated the accuracy of different SNP detection tools on exonic regions. The performance of different SNP callers on exonic regions is shown in Supplementary Table S8. On each dataset, NanoSNP and Clair3 have significantly higher accuracies than Clair, Pepper-DeepVariant and NanoCaller. NanoSNP has the highest precisions and second highest recalls and F1 scores. Clair3 has the highest recalls and F1 scores. In each dataset, the F1 score of NanoSNP is close to that of Clair3. The difference between the F1 scores of NanoSNP and Clair3 is not more than 0.003.

On the cross-genome testing of the low-coverage Nanopore sequencing data, Clair3 and NanoSNP outperform other SNP callers including Clair, Pepper-DeepVariant and NanoCaller on all six genomes. NanoSNP achieves the highest precision and second highest recall and F1 score in five SNP callers. Since NanoSNP is trained on HG001 genome, the Ashkenazim Trio and Chinese Trio genomes are new to NanoSNP. The SNP calling performance indicates that NanoSNP has good robustness for SNP identification on low-coverage Nanopore sequencing data in real-world scenarios.

### 3.4 SNP-calling performance in difficult-to-map regions

GA4GH and GIAB have defined the difficult-to-map regions on the reference genome GRCh38. The difficult-to-map regions are the union of all tandem repeats, all homopolymers >6 bp, all imperfect homopolymers >10 bp, all difficult to map regions, all segmental duplications, GC <25 or >65%, bad promoters, and other difficult regions such as major histocompatibility complex (MHC). We tested the SNP calling performance of several SNP callers in difficult-to-map regions on Nanopore reads of six genomes HG002–HG007. The coverage of each dataset is around 16×.

We downloaded the BED files of difficult-to-map regions from the public website. Each difficult-to-map region on the reference is recorded as an interval including the start position and end position. The benchmark datasets are GIAB variants dataset v3.3.2 of each genome. Each benchmark contains two files, one is a VCF file that records variant sites, and the other is a BED file that records high-confidence regions for SNP detection. The SNP sites that fall in the high-confidence regions in the VCF file have higher confidence than the SNP sites in other regions. We used Bedtools to obtain the intersection of the difficult-to-map regions and the high-confidence regions for each human genome. In the genomes HG002, HG003 and HG004 of Ashkenazim Trio, SNPs in the difficult regions account for about 18% of total SNPs in the high-confidence regions. In the genome HG005, HG006 and HG007 of Chinese Trio, the percentage is around 15%. These proportions indicate that SNPs in the difficult-to-map regions account for a significant fraction of all the SNPs in the high-confidence regions.

The evaluation of SNPs identified by NanoSNP and the compared SNP callers in the difficult-to-map regions is shown in Table 1. In the difficult-to-map regions on GRCh38, NanoSNP has the highest precisions and F1 scores in all the SNP callers on each dataset of Ashkenazim Trio and Chinese Trio. On the datasets of genomes HG002, HG003, HG004, the F1 scores of NanoSNP are 93.1%, 93.4% and 92.6% and the F1 scores of Clair3 are 92.8%, 93.0% and 92.5%. The F1 scores of other SNP callers are much lower than the F1 scores of NanoSNP and Clair3. On the dataset of genomes HG005, HG006 and HG007, the F1 scores of NanoSNP are 92.7%, 93.4% and 93.2%. The F1 scores of Clair3 are 92.3%, 93.0% and 92.8%.

**Table 1.** Evaluation of SNP calls identified by the different SNP calling methods in the difficult-to-map regions on the low-coverage (16×) Nanopore sequencing datasets.

Metrics	Methods	HG002	HG003	HG004	HG005	HG006	HG007
Precision	Clair	0.892	0.897	0.890	0.854	0.880	0.880
	Clair3	0.916	0.916	0.911	0.901	0.912	0.911
	Pepper-DeepVariant	0.881	0.868	0.860	0.850	0.862	0.856
	NanoCaller	0.659	0.672	0.651	0.603	0.660	0.647
	NanoSNP	**0.935**	**0.935**	**0.926**	**0.925**	**0.933**	**0.933**
Recall	Clair	0.782	0.796	0.782	0.780	0.792	0.788
	Clair3	**0.941**	**0.944**	**0.940**	**0.946**	**0.949**	**0.945**
	Pepper-DeepVariant	0.899	0.906	0.897	0.898	0.910	0.904
	NanoCaller	0.902	0.906	0.898	0.895	0.906	0.900
	NanoSNP	0.928	0.932	0.926	0.929	0.936	0.930
F1 score	Clair	0.834	0.843	0.833	0.815	0.834	0.832
	Clair3	0.928	0.930	0.925	0.923	0.930	0.928
	Pepper-DeepVariant	0.890	0.887	0.878	0.874	0.885	0.879
	NanoCaller	0.761	0.772	0.755	0.721	0.764	0.753
	NanoSNP	**0.931**	**0.934**	**0.926**	**0.927**	**0.934**	**0.932**

The bold in the table means the best results.

The evaluation of SNP calls identified by several SNP callers in the MHC regions is shown in Supplementary Table S9. NanoSNP and Clair3 have higher accuracies than Clair, Pepper-DeepVariant and NanoCaller. NanoSNP achieves the highest precisions on all the genomes and Clair3 has the highest F1 scores. On the Ashkenazim Trio (HG002, HG003 and HG004), the F1 scores of NanoSNP are 95.9%, 97.5% and 97.9%, and the F1 score of Clair3 are 96.4%, 97.8% and 98.0%. On the Chinese Trio, the F1 scores of NanoSNP are 96.8%, 97.3% and 96.4%. The F1 scores of Clair3 are 97.3%, 98.0% and 97.4%. The F1 scores of Clair3 are slightly higher than that of NanoSNP. Overall, NanoSNP and Clair3 have similar SNP identification performance in difficult-to-map regions and MHC regions, and both are significantly better than other compared SNP callers.

### 3.5 Performance of pileup model and haplotype model

When the coverage of Nanopore sequencing reads is insufficient, it is difficult to determine whether the difference between the read base and reference base is caused by sequencing error or SNP. In NanoSNP, we employed two different models to identify SNP sites sequentially. The pileup model first detected a subset of SNP sites based on the pileup image of aligned reads. This set of SNPs is phased by WhatsHap and used for the phasing of Nanopore reads. We then generated the haplotype image and the pileup image of each haplotype and used the haplotype model to identify SNPs. We evaluated the SNPs called by the pileup model and the haplotype model on the low-coverage Nanopore sequencing of genomes HG002–HG007.

The evaluations of SNPs identified by the two models in NanoSNP are shown in Supplementary Table S10. The precision, recall and F1 score of the pileup model on six human datasets are 90.3%, 93.6% and 91.9% on average. The precision, recall and F1 score of the haplotype model on six human datasets are 97.2%, 96.5% and 96.9% on average. The performance of the haplotype model has a significant improvement compared to that of the pileup model. Especially on the genome HG005, the precision, recall, F1 scores of the haplotype model get improved by around 11.0%, 3.4% and 7.2% than that of the pileup model. The improvement can indicate that phasing Nanopore reads benefits the SNP identification on low-coverage Nanopore sequencing data.

### 3.6 Computational setup and runtime

To assess the runtime of NanoSNP, we divided the pipeline of NanoSNP into four main steps, including feature generation of the pileup model, prediction of the pileup model, feature generation of the haplotype model and prediction of the haplotype model. The prediction of the pileup model and the haplotype model ran on a graphics processing unit (GPU), and the feature generation ran on a central processing unit (CPU). We ran NanoSNP and three other SNP callers on the server with 40 CPU processors, an Nvidia Tesla A100 GPU. For human whole-genome sequencing data with 16× sequencing depth, NanoSNP took a total of 16 h, where each main step took 4, 2, 7 and 3 h, respectively. We then ran NanoCaller, Pepper-DeepVariant, Clair, Clair3 to identify the SNPs under the same computing resources. The runtime of these four tools is around 8, 8, 9 and 10 h, respectively. In SNP calling, NanoSNP has two identification steps, including calling SNPs with the pileup information and calling SNPs with the haplotype information. Compared with the methods based on either pileup information (Clair) or haplotype information (NanoCaller), NanoSNP has one more identification step. Therefore, the running time of NanoSNP was nearly 50% longer than that of the compared SNP callers. Compare to Pepper-DeepVariant, the second step of NanoSNP verifies all the candidate SNP sites, which spends more computing time. Compare to Clair3, the full-alignment model in Clair3 directly takes phased local alignment as a feature image, while NanoSNP calculates both local features and long-range features of candidate SNP sites from reads alignment, which increases the runtime of NanoSNP.

## 4 Conclusion

On the low-coverage Nanopore sequencing dataset, it is difficult to determine whether the difference between the read base and reference base is a sequencing error or SNP due to the high sequencing error rate and insufficient sequencing coverage. This study proposed a novel deep learning-based SNP caller, NanoSNP for low-coverage Nanopore sequencing data. In NanoSNP, we design a two-step SNP identification workflow. In the first step, the pileup model of NanoSNP takes the pileup feature of aligned reads to identify a set of SNPs. This set of SNPs is used for phasing aligned reads, which divides the sequencing reads into different haplotypes. In the second step, the haplotype model of NanoSNP extracts the long-range haplotype information and short-range pileup information of each haplotype. The haplotype model can correct part of the false SNP calls of the pileup model and identifies SNPs that are missed by the pileup model.

We evaluate several Nanopore SNP callers on low-coverage Nanopore sequencing reads with different coverage values (16×, 19×, 22× and 25×). NanoSNP performs better than Clair, Pepper-DeepVariant and NanoCaller. When the coverage is greater than 16×, the F1 scores of NanoSNP are close to that of Clair3. In the cross-genome testing on six human genomes HG002–HG007, NanoSNP achieves the highest precision, second highest recall and F1 score on each low-coverage Nanopore dataset. Usually, the SNP identification in the difficult-to-map regions and the MHC regions of the human genome is more difficult and error-prone. By benchmarking the SNPs in these regions, we find that NanoSNP has a good performance in the difficult-to-map regions and the MHC regions.

NanoSNP is designed for identifying SNPs on low-coverage Nanopore sequencing data. NanoSNP cannot identify SNPs with PacBio reads and Illumina reads. We will further develop NanoSNP to support multiple sequencing platforms in future work.

## Supplementary Material

btac824_Supplementary_Materials
