# Supplementary material for: NanoSNP: a progressive and haplotype-aware SNP caller on low-coverage nanopore sequencing data
Source: Bioinformatics. 2022 Dec 22;39(1):btac824. doi: 10.1093/bioinformatics/btac824 (PMC9822538; doi:10.1093/bioinformatics/btac824)
Supplement: btac824_Supplementary_Materials [file btac824_supplementary_materials.pdf]

Supplementary Materials

Supplementary Texts

Text S1. Commands and training dataset of tools

Command of Clair (v2.0.6)

```
python clair.py callVarBamParallel \
--chkpnt_fn ont/model \
--ref_fn reference.fna \
--bam_fn input.bam \
--threshold 0.2 \
--sampleName sample \
--output_prefix output_dir/output > command.sh
cat command.sh | parallel -j10
for i in output.*.vcf; do if ! [ -z "$(tail -c 1 "$i")" ]; then echo "$i"; fi ; done | grep -f -command.sh | sh
vcfcat output*.vcf | bcftools sort -m 2G | bgzip tabix snp_and_indel.vcf.gz
bcftools filter -s LowQual -e '%QUAL<748' -Oz snp_and_indel.vcf.gz > snp_and_indel.filter.vcf.gz
```

Training dataset of Clair: ONT R9.4.1, Guppy 4.2.2, HG001,2

Command of NanoCaller (v1.0.1)

```
singularity run --nv \
nanocaller_1.0.1.sif \
python NanoCaller_WGS.py \
-bam input.bam \
-ref reference.fna \
-o output_dir \
-cpu threads \
-p ont
```

Training dataset of NanoCaller: ONT R9.4.1, Guppy 4.2.2, HG001

Command of Pepper-DeepVariant (r0.6)

```
singularity run --nv \
pepper_deepvariant_r0.6.sif \
run_pepper_margin_deepvariant call_variant \
-b input.bam \
-f reference.fna \
-o output_dir \
-t threads \
--ont_r9_guppy5_sup
```

Training dataset of Pepper-DeepVariant: ONT R9.4.1, Guppy 4.2.2, HG002

Command of Clair3 (v0.1-r8)

```
source activate clair3
bash run_clair3.sh \
--bam_fn=input.bam \
--ref_fn=reference.fna \
--threads=threads \
--platform=ont \
--model_path=ont_model \
--output=output_dir
```

Training dataset of Clair3: ONT R9.4.1, Guppy 4.2.2, HG001

Command of NanoSNP (v2.1)

```
singularity exec --nv --containall nanosnp_v2.1-gpu.sif run_caller.sh \
-b input.bam \
-f reference.fna \
-t threads \
-c coverage \
-o output_dir
```

Training dataset of NanoSNP: ONT R9.4.1, Guppy 4.2.2, HG001

Command of Hap.py (v0.3.12)

```
singularity exec happy_docker_image.sif \
/opt/hap.py/bin/hap.py \
GIAB_benchmark.vcf.gz \
input.vcf.gz \
-f GIAB_benchmark.bed \
-r reference.fna \
-o output_dir \
--pass-only \
--engine=vcfeval \
--threads=threads
```

Command of Minimap2 (v2.17-r941)

```
minimap2 -ax map-ont -t 48 reference.fna reads.fastq.gz > align.sam
samtools view -bS -@48 align.sam > align.bam
samtools sort -@48 align.bam > align.sort.bam
samtools index -@48 align.sort.bam
```

Text S2. The cluster of FP/FN SNP calls by NanoSNP

We calculate the distance of adjacent FP calls or adjacent FN calls. For two adjacent FP calls, if the distance is smaller than 200bp, we put them into the same cluster. Finally, if the cluster has more than 20 FP calls, we say that these FP calls are clustered in this region. For two adjacent FN calls, if the distance

is smaller than 1000bp, we put the two FN calls into the same cluster. Finally, if the cluster has more than 20 FN calls, we say that these FN calls are clustered in this region. On the 16X HG002 dataset, we find several clusters of FP calls and FN calls. The IGV visualization of one FP calls cluster is shown in **Figure S1**. In the region of chr13:63,004,608-63,110,069, there are a total 670 FP calls, the average distance between two FP calls is 157bp. The read coverage of this region (53x) is three times higher than the average depth (16x). Then we check the region with the UCSC Genome Browser, we find that the reason for the clustering of FP sites is that there is a large duplication in this region. The existence of duplication causes reads from other regions to align to this region, which results in the FP SNPs. **Figure S2** shows a region enriched for FN sites. In the region of chr7:147,307,750-147,328,816, there is total of 123 FN SNP calls. The average distance between two adjacent FN calls is 171bp. In this region, there is no large duplication. However, the reads coverage of this region is lower than other surrounding regions and the average coverage of the region is less than 10.

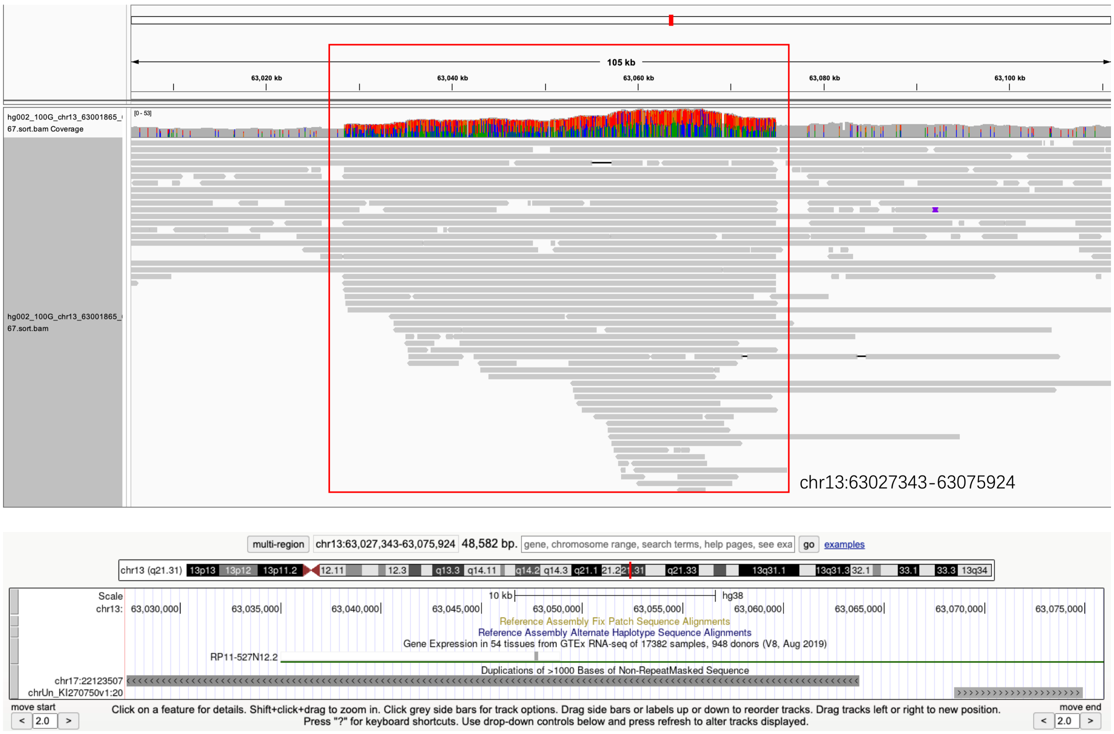

Figure S1. IGV visualization of FP calls cluster.

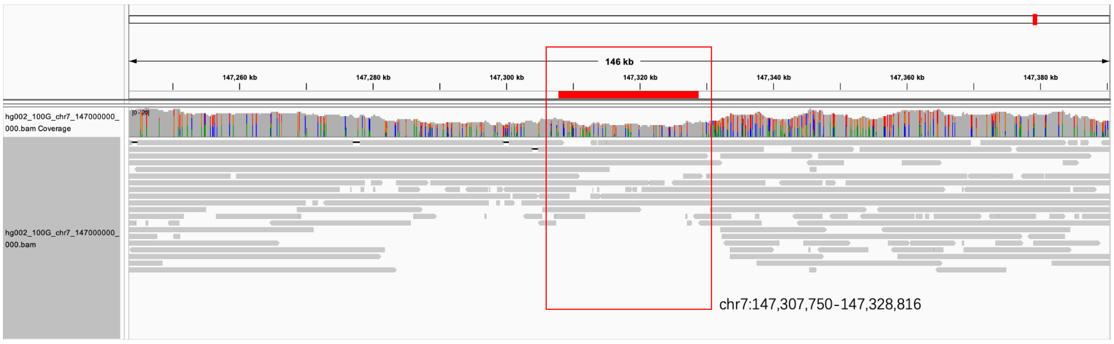

Figure S2. IGV visualization of FN calls cluster.

Text S3. Coverage distributions at TP/FP/FN sites

We count the read coverage at TP/FP/FN SNP sites predicted by different SNP callers on the dataset of 16X HG002 reads. We then plot the probability density distribution of read coverage for TP, FP, and FN sites. From **Figure S3**, we can see that for the predictions of NanoSNP, the coverage of FN sites is lower than that of TP and FP sites, and the coverage of TP sites is the highest. The coverage distributions of Clair SNP calls and Clair3 SNP calls are similar to that of NanoSNP calls. For the coverage distribution of Pepper-DeepVariant SNP calls, the coverages of FP and FN sites are close, and both are less than that of TP sites. For the coverage distribution of NanoCaller SNP calls, the coverages of TP and FN sites are close, mainly concentrated between 10X and 25X. However, the coverage of FP sites is widely distributed. Many FP sites have very low coverage or very high coverage.

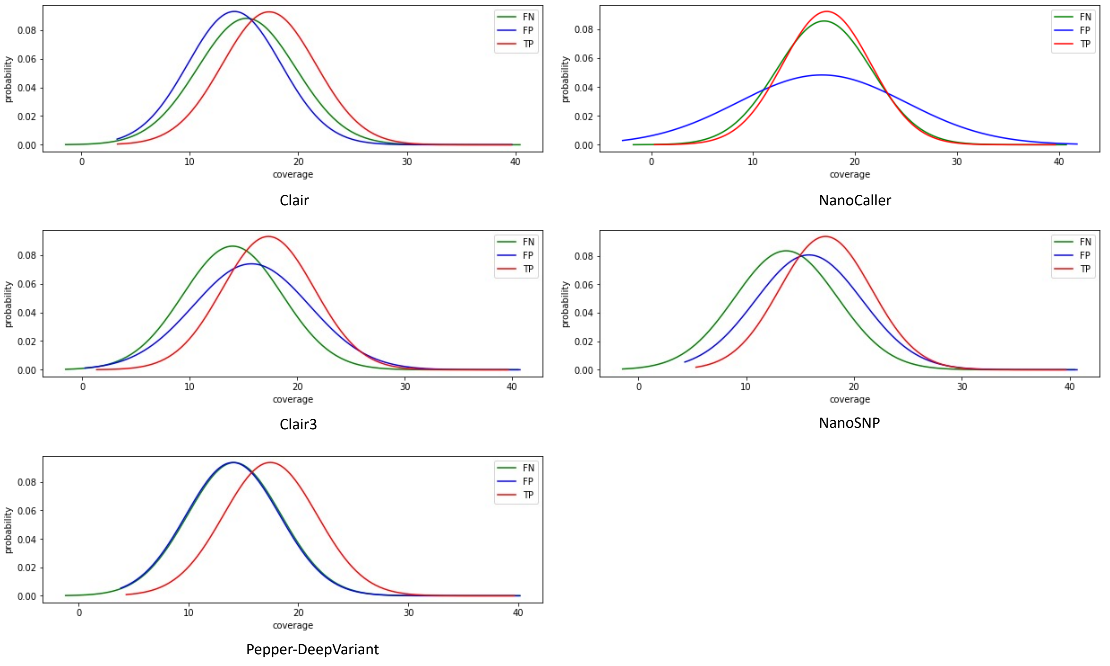

Figure S3. Coverage distribution at TP/FP/FN sites predicted by different SNP callers.

Text S4. IGV screenshots of read alignment and output VCFs

In the SNP calls of the dataset of 16X HG002, NanoSNP identifies an SNP site at the position of chr1:148635790, while all other SNP callers miss this site. According to the benchmark of GIAB, there exists an SNP at this position. The reads alignment at this position is shown in **Figure S4**. The read coverage at the current position is 17. The reference base is "T" and the base of the alternate allele is "G". The frequency of the alternate allele equals 0.176. The genotype and the zygosity of NanoSNP prediction are "T,G" and "0/1" respectively.

According to the GIAB benchmark, the position chr7: 100965939 of the HG002 genome is a non-SNP position. However, Clair, Clair3, Pepper-DeepVariant and NanoCaller all consider this site to be a heterozygous SNP site. NanoSNP considers this site to be a non-SNP site. The reads alignment of this position is presented in **Figure S5**, the read coverage of this position is 20. The reference base is "T" and the base of the alternate allele is "G". The frequency of the alternate allele equals 0.4.

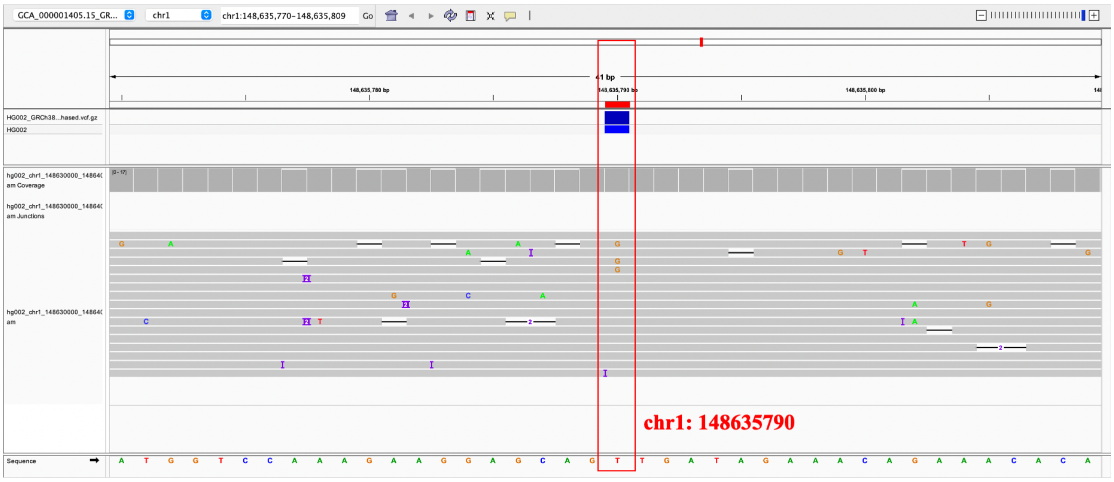

Chrom	Position	REF	ALT	Genotype	NanoSNP	Clair	Clair3	Pepper-DV	NanoCaller
chr1	148635790	T	G	0|1	T,G	FN	FN	FN	FN

Figure S4. IGV of reads alignment at chr1: 148635790 on the dataset of 16X HG002

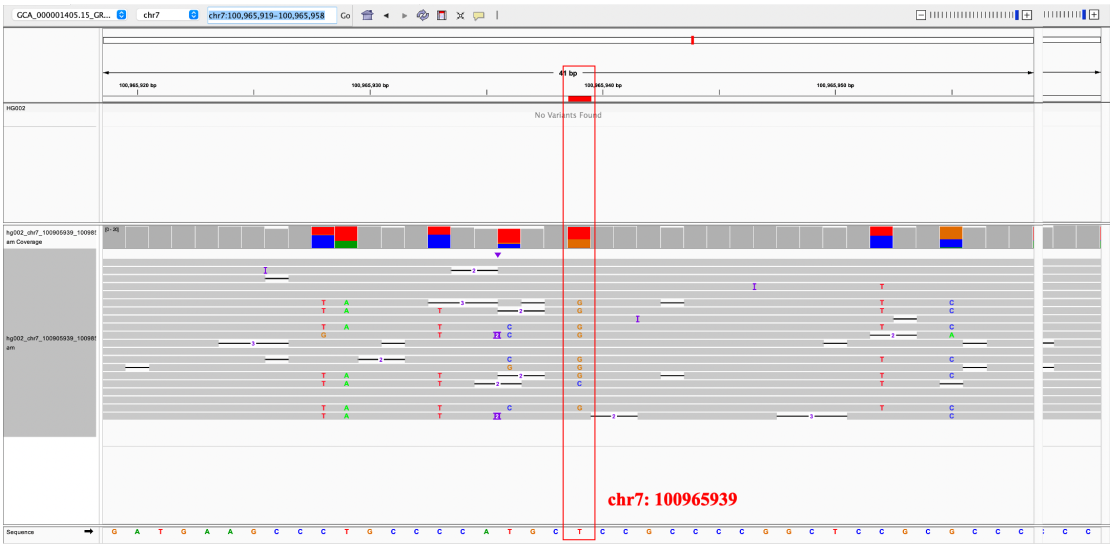

Chrom	Position	REF	ALT	Genotype	NanoSNP	Clair	Clair3	Pepper-DV	NanoCaller
chr7	100965939	T	T	0|0	T,T	T,G	T,G	T,G	T,G

Figure S5. IGV of reads alignment at chr7: 100965939 on the dataset of 16X HG002

Text S5. Comparison of SNP calls by NanoSNP and Clair3

We compare the SNP calls of NanoSNP and Clair3 on the dataset of 16X HG003 in **Figure S6**. From TP calls, NanoSNP detects 2,787,334 SNP sites, while Clair3 identifies 2,814,261 SNP sites. NanoSNP identifies 13,826 unique SNPs, while Clair3 identifies 40,753 unique SNPs. From FP calls, NanoSNP has 33,215 unique FP SNPs, while Clair3 has 53,456 unique FP SNPs. From FN calls, NanoSNP misses 36,885 unique SNP sites, while Clair3 misses 8624 unique SNP sites. From the comparison, we can see that Clair3 can detect more SNP sites than NanoSNP. However, this also increases the error of FP.

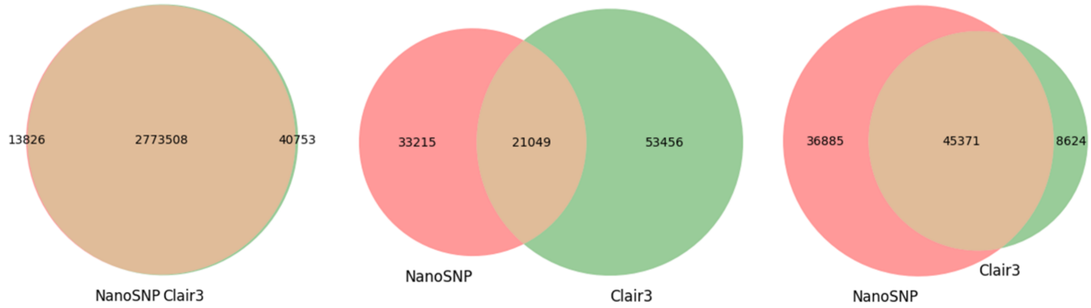

Figure S6. Comparison of SNP calls by NanoSNP and Clair3 on 16X HG003 dataset. The left figure presents the TP SNP calls by NanoSNP and Clair3. The middle figure presents the FP SNP calls and the right figure presents the FN SNP calls.

Text S6. Evaluation of different SNP callers on the dataset of mixtures of haploid bacterial

We evaluated different SNP callers on a dataset of a mixture of Nanopore sequencing data of two *Klebsiella pneumoniae* samples (NCBI ID: ERR2797014 and ERR2797015). The ONT reads of each *K. pneumoniae* sample were sampled to 16X coverage. We used minimap2 to align the sequencing reads to the reference genome *K. pneumoniae* subsp. pneumoniae HS11286 (NCBI ID: NC_016845.1). Then, Clair, Clair3, Pepper-DeepVariant, NanoCaller, and NanoSNP were used to call SNP sites according to the alignment bam file. Since Clair output did not contain any SNP sites, we did not include Clair in the comparison. To evaluate the accuracy of SNP calls, we downloaded the assembly of each *K. pneumoniae* sample and aligned the assembly to reference genome using MUMmer. The union of the SNP sites reported by MUMmer was used as a benchmark. The SNP calls of different SNP callers on the dataset of mixtures of haploid bacterial are shown in **Figure S7.** NanoSNP achieves the highest Recall and NanoCaller achieves the highest Precision and F1 score. The F1 scores of Clair3, Pepper-DeepVariant, NanoCaller and NanoSNP are 48.08%, 44.31%, 51.48% and 47.19%.

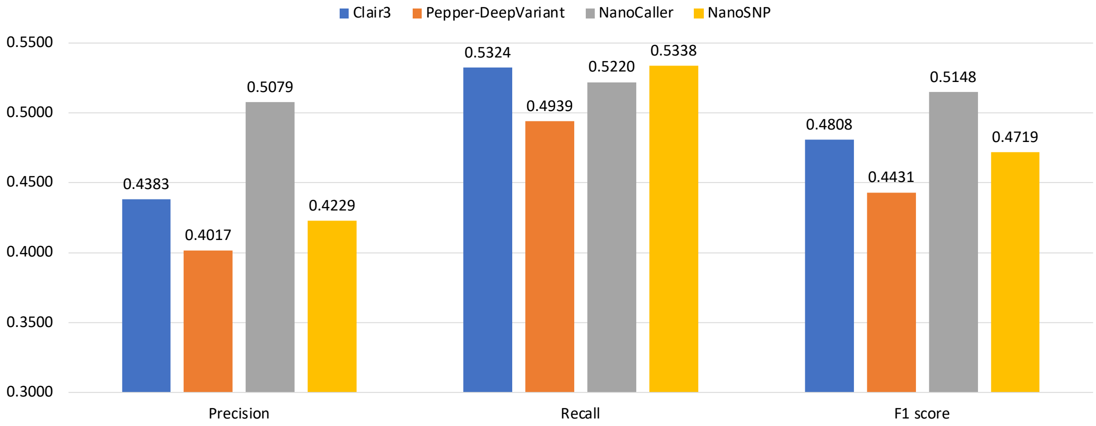

Figure S7. Evaluation of Clair3, Pepper-DeepVariant, NanoCaller and NanoSNP on Klebsiella pneumoniae dataset.

Text S7. Fraction of final SNPs chosen by the pileup model and haplotype model of NanoSNP

The pileup model of NanoSNP takes the pileup feature of aligned reads to identify SNPs. This model can identify most of the SNP sites in the dataset, and these SNP sites have significant

SNP information. The haplotype model of NanoSNP extracts the long-range haplotype information and short-range pileup information to detect SNPs. The haplotype model can correct part of the false SNP calls of the pileup model and identifies SNPs that are missed by the pileup model. In the SNP calls of dataset 16X HG002, the total number of SNPs is 3967,853. 71.9% (2,854,785) of SNPs are called by the pileup model, and 28.1% (1,113,068) of SNPs are called by the haplotype model.

Text S8. Correlation between the VAFs of SNPs identified by NanoSNP and the true VAFs

NanoSNP calculates the VAFs for identified SNPs and writes the VAFs to the output VCF file. In NanoSNP, each identified SNP has a variant allele fraction, which is the relative frequency of a variant allele. The correlation between the VAFs of SNPs identified by NanoSNP and the true VAFs equals 66.9% on the dataset of 16X HG002. The VAF of SNPs identified by NanoSNP is correlated with the number of paternal reads, the number of maternal reads, and the sequencing error rate at the current position. We collected the VAFs in the TP SNPs detected by NanoSNP on the dataset of 16X HG002, the minimum variant allele fraction is 4.8%.

Supplementary Tables

Table S1. Evaluation of different quality thresholds (ϵ) of high-quality heterozygous SNPs generated by the pileup model. The dataset is from HG003 with coverage 16X.

Quality threshold (ϵ)	TP	FN	FP	Precision	Recall	F1 score
$\epsilon = 12$	2787208	95469	69742	0.976	0.967	0.971
$\epsilon = 14$	2787634	95643	70006	0.967	**0.977**	**0.972**
$\epsilon = 16$	2787208	95469	70130	0.967	0.975	0.971
$\epsilon = 18$	2786212	96465	70078	0.967	0.975	0.971

Table S2. Evaluation of pileup feature and haplotype feature for the haplotype model in NanoSNP. The dataset is from HG003 with coverage 16X.

Features in haplotype model	TP	FN	FP	Precision	Recall	F1-score
Pileup feature alone	**2790284**	**90393**	93162	0.968	0.**969**	0.968
Haplotype feature alone	2789309	93368	88596	0.969	0.968	0.968
Pileup feature + haplotype feature	2787334	95343	**70006**	**0.976**	0.967	**0.971**

Table S3. Evaluation of different values of quality threshold $\boldsymbol{\delta}$ to merge the SNP calls of the pileup model and the SNP calls of the haplotype model in NanoSNP. The dataset is from HG003 with coverage 16X.

Quality threshold (δ)	TP	FN	FP	Precision	Recall	F1-score
$\delta = 10$	**2793641**	**89036**	89004	0.969	**0.969**	0.969
$\delta = 11$	2792362	90315	82378	0.971	**0.969**	0.970
$\delta = 12$	2790347	92330	76077	0.973	0.968	0.971
$\delta = 13$	2787334	95343	70006	0.976	0.967	0.971
$\delta = 14$	2783250	99427	63820	0.978	0.966	**0.972**
$\delta = 15$	2777306	105371	57556	0.980	0.963	**0.972**
$\delta = 16$	2768640	114037	50912	0.981	0.960	0.971
$\delta = 17$	2755503	127174	43356	0.985	0.956	0.970
$\delta = 18$	2731288	151389	34459	0.988	0.947	0.967
$\delta = 19$	2661636	221041	**22412**	**0.991**	0.923	0.956

Table S4. Detail of sequencing datasets and benchmark variants datasets.

Sample	Coverage	Basecaller	Accession	Benchmark	Reference
HG001	88.33	Guppy 4.2.2	HG001_NBT2018_Guppy_4.2.2.fastq.gz<br>HG001_Circulomics_Guppy_4.2.2.fastq.gz	GIAB NIST v3.3.2	GRCh38
HG002	51.06	Guppy 4.2.2	HG002_GIAB_MinION_GridION_Guppy_4.2.2.fastq.gz<br>HG002_GIAB_PromethION_Guppy_4.2.2_prom.fastq.gz	GIAB NIST v3.3.2	GRCh38
HG003	78.94	Guppy 4.2.2	GM24149_1_Guppy_4.2.2_prom.fastq.gz<br>GM24149_2_Guppy_4.2.2_prom.fastq.gz<br>GM24149_3_Guppy_4.2.2_prom.fastq.gz	GIAB NIST v3.3.2	GRCh38
HG004	81	Guppy 4.2.2	GM24143_1_Guppy_4.2.2_prom.fastq.gz<br>GM24143_2_Guppy_4.2.2_prom.fastq.gz<br>GM24143_3_Guppy_4.2.2_prom.fastq.gz	GIAB NIST v3.3.2	GRCh38
HG005	51.97	Guppy 4.2.2	01_09_20_R941_GM24631_1_Guppy_4.2.2_prom.fastq.gz<br>01_09_20_R941_GM24631_2_Guppy_4.2.2_prom.fastq.gz<br>01_09_20_R941_GM24631_3_Guppy_4.2.2_prom.fastq.gz	GIAB NIST v3.3.2	GRCh38
HG006	93.33	Guppy 4.2.2	01_09_20_R941_GM24694_1_Guppy_4.2.2_prom.fastq.gz<br>01_09_20_R941_GM24694_2_Guppy_4.2.2_prom.fastq.gz<br>01_09_20_R941_GM24694_3_Guppy_4.2.2_prom.fastq.gz	GIAB NIST v3.3.2	GRCh38
HG007	76.06	Guppy 4.2.2	01_09_20_R941_GM24695_1_Guppy_4.2.2_prom.fastq.gz<br>01_09_20_R941_GM24695_2_Guppy_4.2.2_prom.fastq.gz<br>01_09_20_R941_GM24695_3_Guppy_4.2.2_prom.fastq.gz	GIAB NIST v3.3.2	GRCh38

Table S5. Evaluation of SNP calls in non-diploid regions by Clair, Clair3, Pepper-DeepVariant, NanoCaller, and NanoSNP.

Models	Total	TP	FN	FP	Recall	Precision	F1_Score
Clair	2422	2102	320	207	0.868	0.910	0.889
Clair3	2422	**2355**	**67**	48	**0.972**	0.980	**0.976**
Pepper-DeepVarnat	2422	2247	175	154	0.928	0.936	0.932
NanoCaller	2422	2281	141	715	0.942	0.761	0.842
NanoSnp	2422	2342	80	**45**	0.967	**0.981**	0.974

Table S6. Performance (precision, recall, F1 score) of SNP calling on different coverages of low-coverage Nanopore sequencing datasets of genome HG003.

Metrics	Methods	10X	13X	16X	19X	22X	25X
Precision	Clair	0.790	0.909	0.909	0.933	0.948	0.958
	Clair3	0.922	0.956	0.972	0.980	0.984	0.986
	Pepper-DeepVariant	0.677	0.816	0.915	0.959	0.976	0.983
	NanoCaller	0.421	0.566	0.733	0.850	0.908	0.937
	NanoSNP	**0.940**	**0.959**	**0.976**	**0.982**	**0.985**	**0.987**
Recall	Clair	0.649	0.877	0.877	0.918	0.941	0.954
	Clair3	**0.902**	**0.956**	**0.976**	**0.985**	**0.989**	**0.991**
	Pepper-DeepVariant	0.806	0.896	0.941	0.966	0.978	0.985
	NanoCaller	0.697	0.887	0.944	0.963	0.971	0.976
	NanoSNP	0.805	0.891	0.967	0.980	0.986	0.989
F1 score	Clair	0.713	0.893	0.893	0.926	0.945	0.956
	Clair3	**0.912**	**0.956**	**0.974**	**0.982**	**0.987**	**0.989**
	Pepper-DeepVariant	0.736	0.854	0.928	0.963	0.977	0.984
	NanoCaller	0.525	0.691	0.826	0.903	0.939	0.956
	NanoSNP	0.867	0.924	0.971	0.981	0.986	0.988

Table S7. Evaluation of SNP calls on different references by NanoSNP.

Dataset	Reference	TP	FN	FP	Precision	Recall	F1_Score
HG002	GRCh38	**2926480**	102878	71085	0.976	0.966	0.971
	GRCh37	2915021	**97229**	**66430**	**0.978**	**0.968**	**0.973**
HG003	GRCh38	2787334	**95343**	70006	0.976	0.967	0.971
	GRCh37	**2915021**	97229	**66430**	**0.978**	**0.968**	**0.973**

Table S8 Evaluation of SNP calls on exonic regions by Clair, Clair3, Pepper-DeepVariant, NanoCaller, and NanoSNP.

Metrics	Methods	HG002	HG003	HG004	HG005	HG006	HG007
Precision	Clair	0.871	0.895	0.882	0.826	0.859	0.860
	Clair3	0.963	0.968	0.965	0.949	0.957	0.958
	Pepper-DeepVariant	0.901	0.913	0.903	0.872	0.878	0.864
	NanoCaller	0.588	0.669	0.628	0.527	0.589	0.572

	NanoSNP	**0.968**	**0.973**	**0.968**	**0.955**	**0.964**	**0.964**
Recall	Clair	0.853	0.884	0.875	0.844	0.859	0.855
	Clair3	**0.972**	**0.978**	**0.976**	**0.972**	**0.977**	**0.974**
	Pepper-DeepVariant	0.922	0.941	0.935	0.917	0.932	0.927
	NanoCaller	0.935	0.945	0.939	0.924	0.934	0.927
	NanoSNP	0.960	0.970	0.966	0.958	0.964	0.961
F1 score	Clair	0.862	0.889	0.878	0.835	0.859	0.857
	Clair3	**0.967**	**0.973**	**0.970**	**0.960**	**0.967**	**0.965**
	Pepper-DeepVariant	0.912	0.927	0.918	0.894	0.904	0.894
	NanoCaller	0.722	0.784	0.753	0.671	0.723	0.708
	NanoSNP	0.964	0.971	0.967	0.957	0.964	0.962

Table S9. Performance of SNP calling in the Major Histocompatibility Complex (MHC) regions by SNP caller Clair, Clair3, Pepper-DeepVariant, NanoCaller, and NanoSNP.

Metrics	Methods	HG002	HG003	HG004	HG005	HG006	HG007
Precision	Clair	0.878	0.920	0.920	0.904	0.913	0.804
	Clair3	0.968	0.978	0.977	0.974	**0.979**	**0.974**
	Pepper-DeepVariant	0.887	0.954	0.958	0.933	0.913	0.916
	NanoCaller	0.702	0.810	0.816	0.750	0.766	0.746
	NanoSNP	**0.976**	**0.981**	**0.981**	**0.975**	**0.979**	**0.974**
Recall	Clair	0.814	0.868	0.890	0.839	0.844	0.905
	Clair3	**0.960**	**0.977**	**0.982**	**0.972**	**0.980**	**0.975**
	Pepper-DeepVariant	0.899	0.930	0.956	0.929	0.946	0.926
	NanoCaller	0.909	0.952	0.965	0.935	0.951	0.911
	NanoSNP	0.943	0.969	0.977	0.961	0.968	0.954
F1 score	Clair	0.845	0.893	0.905	0.870	0.877	0.852
	Clair3	**0.964**	**0.978**	**0.980**	**0.973**	**0.980**	**0.974**
	Pepper-DeepVariant	0.893	0.942	0.957	0.931	0.929	0.921
	NanoCaller	0.792	0.875	0.884	0.832	0.848	0.820
	NanoSNP	0.959	0.975	0.979	0.968	0.973	0.964

Table S10. Evaluation of the pileup model and the haplotype model on the low-coverage (16X) Nanopore sequencing.

Metrics	Models	HG002	HG003	HG004	HG005	HG006	HG007
Precision	pileup model	0.916	0.917	0.908	0.870	0.900	0.904
	pileup model + haplotype model	**0.976**	**0.976**	**0.972**	**0.966**	**0.973**	**0.973**
Recall	pileup model	0.935	0.939	0.933	0.930	0.940	0.936
	pileup model + haplotype model	**0.966**	**0.967**	**0.964**	**0.962**	**0.968**	**0.965**
F1 score	pileup model	0.925	0.928	0.920	0.899	0.920	0.920
	pileup model + haplotype model	**0.971**	**0.971**	**0.967**	**0.964**	**0.970**	**0.969**
